# Human adenovirus load in respiratory tract secretions are predictors for disease severity in children with human adenovirus pneumonia

**DOI:** 10.1186/s12985-018-1037-0

**Published:** 2018-08-07

**Authors:** Leyun Xie, Bing Zhang, Jieying Zhou, Han Huang, Saizhen Zeng, Qin Liu, Zhiping Xie, Hanchun Gao, Zhaojun Duan, Lili Zhong

**Affiliations:** 1 Hunan Provincial People’s Hospital, No 61, Jie-Fang West Road, Changsha, 410005 People’s Republic of China; 2 Key Laboratory for Medical Virology, Ministry of Health, National Institute for Viral Disease Control and Prevention, China CDC, Beijing, 102206 People’s Republic of China

**Keywords:** HAdV, Pneumonia, Children, HAdV- load, Risk factor

## Abstract

**Background:**

Pneumonia is a serious public health issue and is concerned around the world. This study is to investigate the association between viral load in children with human adenovirus (HAdV) pneumonia and disease severity.

**Methods:**

A total of 1313 cases of children hospitalized in Hunan Provincial People’s Hospital due to community acquired pneumonia (CAP) from April 2011 to May 2014 were enrolled in this study. Samples of nasopharyngeal aspirate were collected for the cohort. WHO criteria for CAP grading was emerged for pneumonia severity classification. Real-time fluorescence quantitative PCR (qRT-PCR) was used to detect 12 kinds of respiratory viruses. HAdV types were identified by nested PCR. The relationship between HAdV load and severity of disease was there by analyzed.

**Results:**

Finally, 174 cases (174/1313, 13.3%) were positive for HAdV, and HAdV type 7 (HAdV-7) was the main serotype (76/174, 43.7%). Among the 174 cases, 70 cases were with HAdV infection alone and 104 cases were accompanied by other viruses. The patients were divided into mild pneumonia group (*n* = 108 cases) and severe pneumonia group (*n* = 66 cases). HAdV load of children in severe pneumonia group was higher than that in mild pneumonia group. Similar result was obtained in the 70 cases with HAdV infection alone after subgrouping. Relevant factors analysis results showed that severe pneumonia children presented lower onset age, more prone to fever, longer fever time, and longer hospital stay compared with that of mild pneumonia children. Children with HAdV-7 infection developed more frequently severe pneumonia. Multivariate regression analysis showed that HAdV load, age, and fever time were risk factors for pneumonia severity.

**Conclusion:**

The severity of HAdV infection is significantly correlated with viral load and serotype.

## Background

Pneumonia is a serious public health issue and is concerned around the world [1]. In China, pneumonia is the main cause of child hospitalization which brings about increased burden for outpatient clinic [2, 3]. Respiratory virus infections are increasingly recognized as playing a major role in the burden of severe acute respiratory illness in many countries due to expanded global surveillance and improved molecular diagnostic testing [4–7]. It is reported that among children with pneumonia, 4–10% are caused by human adenovirus (HAdV). Liu et al. demonstrated that in China, HAdV are most frequently associated with pediatric severe pneumonia, accounting for 32.2% of severe pneumonia [8]. Additionally, HAdV it is the leading cause of death in severe pneumonia children [8]. Varying degrees of lung sequelae are noted in about 14–60% of HAdV infection caused pneumonia [9].

In recent years, more and more people have recognized the role of adenovirus in severe respiratory infection: resulting in increased hospitalization and mortality [10, 11]. The severity of lower respiratory tract infection caused by HAdV is related to HAdV types, age of onset, immune status, and environment [7, 12, 13]. Notwithstanding children and patients with immunodeficiencies are at increased risk for severe HAdV disease, most children with HAdV requiring hospitalization are previously healthy. In healthy children, most adenovirus respiratory infections are mild and indistinguishable from other viral causes. However, specific serotypes, such as serotypes human adenovirus type 3 (HAdV-3), human adenovirus type 7 (HAdV-7), and human adenovirus type 14 (HAdV-14) have been implicated with fatal outcomes [7, 14, 15]. HAdV-3 and HAdV-7 are the most common types in China, and HAdV-7 is more likely to cause severe pneumonia and significant mortality especially in infants and young children (6–24 months) [10].

Although identification of HAdV genotype or species has significant epidemiologic and clinical importance, most laboratories only routinely perform HAdV detection. Thus far, HAdV surveillance in China is sparse, and some HAdV infections are chiefly identified during outbreak events. Researchers have tried to determine the impact of HAdV on severe pneumonia that requires hospitalization in the country, however, there is a lack of information regarding prevalence, genotypes, and molecular epidemiology of HAdV in China. To this end, in this study we investigated the relationship between viral load and pneumonia manifestations caused by HAdV in hospitalized children by polymerase chain reaction (PCR). We intended to provide a method to pre-judge the disease severity in the populations with high risk of HAdV infection and to facilitate pneumonia treatment.

## Methods

### Subjects

This retrospective study recruited children who were less than 14 years old and hospitalized in Hunan Province People’s Hospital due to pneumonia from April 2011 to May 2014. The children who had evidence of an acute respiratory illness defined as cough, sputum production, dyspnea, tachypnea, abnormal lung examination, or respiratory failure and had evidence consistent with pneumonia assessed within 72 h before or after admission by chest radiography were included into this study. The exclusion criteria of the study were as follows: newborns; infection of HIV; leukemia; known or suspected active tuberculosis; receiving immunosuppressive agents; immunodeficiency; chemotherapy; and chronic conditions (eg, congenital heart disease; chronic lung disease). Positive of two or more kinds of viruses in any combination was defined as co-detection. Clinical data including clinical manifestation, laboratory examination, and diagnostic results were collected through checking the medical records. The study was approved by the Ethics Committee of Hunan Provincial People’s Hospital.

### Samples collection

Nasopharyngeal aspirations (NPAs) of all subjects were obtained within 24 h after admission. About 1–2 ml NPAs were drawn using a disposable sterile suction tube which was inserted 7~ 8 cm into the throat via the nose and under the pharyngeal. NPAs samples were then transferred to sterile collection tube after normal saline was added. Then 2 ml of virus protection solution containing 200 U/ml penicillin, 200 U/ml streptomycin, 200 U/ml amphotericin B, and 0.125% BSA was added into NPAs samples. After mixing, the samples were immediately placed in − 80 °C refrigerator for future examination.

Other microbiological including typical bacteria were detected by Gram staining of sputum specimens and blood culture. Atypical bacteria, chlamydophila pneumoniae and mycoplasma pneumoniae, were detected by blood antigen. All specimens were stored in dry ice at low temperature and transported to the China Center for Disease Control and Prevention for respiratory virus identification.

### Definition of clinical severity

Pneumonia severity classification was performed according to the criteria of the WHO guidelines. Based on the clinical features, pneumonia is classified as very severe, severe, or non-severe, with specific treatment for each of them. Oxygen was given in PICU (Pediatric intensive care unit) to severe and very severe pneumonia patients. Severe cases were identified in the presence of cough or difficult breathing and at least one of the following signs: lower chest wall in drawing; nasal flaring; or grunting (in young infants). In children with a diagnosis of CAP or severe CAP, a diagnosis of very severe CAP was based on the presence of at least one of the following features: central cyanosis; inability to breastfeed, drink, or vomit everything; convulsions, lethargy, or unconsciousness; or severe respiratory distress [16]. In this study the patients were classified into mild pneumonia (non-severe) group and severe pneumonia group.

### HAdV and other respiratory virus monitoring

HAdVs quantitative analysis was performed using NPAs samples by real-time PCR. The viruses included respiratory syncytial virus (RSV), human rhinovirus (HRV), influenza virus A(IFVA), influenza virus B(IFVB), parainfluenza 1–3(PIV1–3), human metapneumovirus (hMPV), human coronaviruses HKU1(HCoV-HKU1), human coronaviruses NL63(HCoV-NL63), and human bocavirus (HBoV). QIAamp columns (QiampMinelute Virus Spin Kit; Qiagen) were chosen to extract DNA from 200 μl of NPAS following the protocol of the manufacturer. The extracted DNA was eluted in 200 μl of distilled water and the eluted solution was conserved at − 20 °C for future use.

Adequate negative controls were set up for monitoring contamination. PCR mixture was made up to a volume of 20 μl, which was consisted of final concentrations of 10 μl Taqman Universal Master MixIIwith UNG, 0.4 μl each primer, 0.4 μl HAdV, 4.8 μl RNA-free water, and 4 μl DNA. Standard curve was generated using the positive control (1 × 10^10^ copies/ml) obtained from the kit. According to the standard curve made by Applied Biosystems (Waltham, MA, USA), the concentration of each sample was calculated. Distilled water was used as negative controls.

### HAdV typing

According to the protocol described by Lu and Erdman, hypervariable region (1–6) of loop 1 of hexon protein were sequenced for HAdV typing [17]. A 688–821-bp fragment of hexon gene was amplified using the AdhexF1/R1 and AdhexF2/R2 primer pairs [17]. GeneAmp 9700 thermal cycler (Applied Biosystems, Foster City, CA) was used to perform the PCR reaction. The conditions of PCR amplification were described as follows: 1 cycle at 94 °C for 3 min; 35 cycles in which each cycle containing 94 °C for 30 s, 52 °C for 30 s, and 72 °C for 60 s; a final extension at 72 °C for 8 min. The products of PCR amplification were determined by electrophoresis with 1% agarose gels and were visualized under UV light with ethidium bromide. Positive HAdV amplification products were sequenced for gene nucleotide at Beijing Tianyi-Huiyuan Bio-science & Technology Inc. (Beijing, China). BLAST comparison was performed between the sequenced results and the sequences in the GeneBank database of U.S. National Center for Biotechnology (NCBI).

### Statistical analysis

Statistical analysis was conducted using SPSS 18.0 (SPSS Inc., Chicago, IL, USA). The *χ*^*2*^ test was used to compare the rates. For comparing the means of two groups, *t* test was chosen if the variance satisfied the normal distribution, and nonparametric Wilcoxon rank sum test was chosen if the variance did not satisfy normal distribution. Logistic regression analysis was used to determine the influencing factors of the severity of the disease, and the sensitivity and specificity of HAdV as a diagnostic were evaluated using the receiver-operator characteristic (ROC) curve. *P* value < 0.05 was considered as statistically significant.

## Results

### Patient characteristics

To learn about the general information of the patients, clinical characteristics of the cohort was studied. Among the 1313 cases enrolled in this study, 824 cases were male and 489 cases were female, and the ratio of male to female was 1.69: 1. The age of all children ranged from 1 month to 13 years. The median age was 11 months (IQR: 5.98–25 months). Children under 5 years old accounted for 91.9% of all cases. The median duration of clinical symptoms before admission was 6 days (interquartile range (IQR): 4–9.5 days). Finally, 179 cases were positive for HAdV. The positive detection rate was 13.3%. Successful HAdV typing was performed in 174 cases. HAdV-7 was the dominant detection type (76/174, 43.7%), followed by HAdV-1 (26/174, 14.9%), HAdV-2 (27/174, 15.5%), HAdV-3 (26/174, 14.9%), HAdV-4 (4/174, 2.3%), HAdV-5(5/174, 2.9%), HAdV-4 (4/174, 2.3%), HAdV-14 (4/174, 2.3%), HAdV-21 (1/174, 0.6%), and HAdV-57 (1/174, 0.6%). Five cases failed for HAdV typing were removed in the following clinical analysis. The age of the 174 HAdV-positive cases ranged from 1 month to 12 years old and the median age was 14 months (IQR: 10–28.5 months). In the 174 HAdV-positive cases, children aged 6 to 23 months were most common (55.8%, 97/174) and male children accounted for 63.8% of the cohort. Other respiratory viruses positive rates were as follows: RSV (34.5%), HRV (17.4%), PIV3 (20.4%), HBOV (18.0%), hMPV (8.4%), IFV (7.8%), PIV1 (2.5%), PIV2 (1.5%), and HCoV (5.6%).

### Clinical manifestations of pneumonia

In order to unravel the influence of HAdV infection on the children, the cohort were subdivided and relative analysis was performed. The detailed results are shown in Table 1. All 1313 pneumonia children were divided into two groups including HAdV infection group and non-HAdV infection group, and they all showed cough. The children under 5 years old were most common in both groups. No significant differences were found in the mean age and sex between the two groups. The children were divided into four age groups: less than 6 months, 6–23 months, 24–60 months, and older than 60 months period. The onset age of the children in the two groups was more frequent in 6–23 months compared with the other 3 groups (*P* < 0.05). For 6–23 months period, the number of children in HAdV infection group was significantly more than that in non-HAdV infection group (55.8% VS 45.7%, *P* = 0.017). For less than 6 months period, the number of children in non-HAdV infection group was significantly higher than that in HAdV infection group (24.9% VS 10.9%, *P* = 0.000). Compared with children in non-HAdV infection group, children in HAdV infection group were more prone to fever (*P* = 0.000) and had longer fever time (*P* = 0.000), were easy to suffer from severe pneumonia (severe *P* = 0.025, very severe *P* = 0.000), admission to PICU (*P* = 0.000), and oxygen requirements (*P* = 0.002), and had longer hospital stay (*P* = 0.046). There were no significant difference in the symptoms of wheezing, vomiting, and diarrhea between the HAdV infection group and non-HAdV infection group.

**Table 1:** Comparison of the clinical characteristics of the different groups of HAdV infection

	HAdV, N(%)					
	Positive (*n* = 174)	Negative (*n* = 1139)	*P*	Single Detection (*n* = 70)^a^	Co-detection (*n* = 104)^b^	*P*
Gender(male)	111(63.79)	713(62.60)	0.98	39(55.71)	68(65.38)	0.199
age (months) IQR	12.5(10–28.25)	11(5.98–25)	0.38	14(11–34.5)	12(8.25–24)	0.063
Age group						
< 6	19(10.92)	284(24.93)	0.000	5(7.14)	14(13.46)	0.190
6–23	97(55.75)	521(45.74)	0.017	37(52.86)	60(57.69)	0.529
24–60	46(26.44)	239(20.98)	0.104	21(30.00)	25(24.04)	0.382
> 60	12(6.90)	95(8.34)	0.517	7(10.00)	5(4.81)	0.228
Duration of Hospitalization (days)	8(6–10)	7(6–9)	0.046	8(6–10)	8(6–10)	0.697
Duration of fever (days)	6(3.5–8)	4(2–6)	0.000	6(4.5–8)	5(3–7.75)	0.202
Cough	174(100)	1139(100)		70(100)	104(100)	
Fever	143(79.89)	693(60.84)	0.000	61(87.14)	80(76.92)	0.092
Diarrhea	33(18.97)	219(19.22)	0.935	13(18.57)	20(19.23)	0.913
Wheezing	73(41.95)	561(49.25)	0.073	24(34.29)	49(47.12)	0.093
Vomiting	30(17.24)	184(16.15)	0.718	11(15.71)	19(18.27)	0.662
Outcome						
Disease severity of CPA						
Mild	108(62.07)	936(82.18)	0.000	38(54.29)	70(67.31)	0.083
Severe	33(18.97)	145(12.73)	0.025	14(20.00)	19(18.27)	0.775
Very severe	33(18.97)	58(5.09)	0.000	18(25.71)	15(14.42)	0.062
Admission to PICU	31(17.80)	89(7.80)	0.000	8(11.40)	23(22.10)	0.071
supplemental oxygen requirement	35(20.10)	134(11.76)	0.002	13(18.57)	22(21.20)	0.877
HAdV load from NPAs(log 10 copies/mL)	3.27 ± 2.15	*N*		3.48 ± 2.44	3.21 ± 2.02	0.408

^a^Seventy patients with single HAdV infection, and mycoplasma, chlamydia, and bacteria were not excluded

^b^One hundred and four patients with HAdV pathogen plus any other respiratory viral pathogen infection, and mycoplasma, chlamydia, and bacteria were not excluded

### Infection of HAdV alone and co-infections of HAdV and other viruses among children with pneumonia

To investigate the difference of HAdV infection alone and co-infections of HAdV and other viruses among children with pneumonia, clinical manifestations were studied and the results are showed in Table 1. Among the 174 HAdV-positive children, 70 children were with infection of HAdV alone and 104 children (59.8%) with co-infected (mixed viral-viral infections). Among all 174 HAdV positive samples, 70 samples (40.2%) were only HAdV positive, while the other 104 samples (59.8%) were co-infected with other viruses. It was noticed that the most common co-infection viruses were RSV (in 46 cases), PIV3 (in 37 cases), HBOV (in 24 cases), HRV (in 20 cases), and HMPV (in 18 cases). The results revealed no significant differences in age, gender, clinical manifestations (fever, duration of fever, duration of hospital stay, wheezing, vomiting, and diarrhea), disease severity, and HAdV load between children with HAdV infection alone and with co-infection of other virus.

### Demographic and clinical characteristics infected children

Of the 1313 children studied, 174 cases were positive for HAdV, including 56 patients with single HAdV pathogen, without blood culture, sputum culture, mycoplasma pneumoniae, chlamydia pneumoniae and any other respiratory viral pathogen infection. Seventy-nine cases were with HAdV and ≥ 1 other viruses detected. Nineteen patients were with ≥1 virus and ≥ 1 typical bacterial pathogen (4 patients with single HAdV plus any typical bacterial pathogen, 15 patients with HAdV plus any other respiratory viral pathogen plus any typical bacterial pathogen). Eighteen were with ≥1 virus and ≥ 1 atypical bacterial pathogen (10 patients with single HAdV plus mycoplasma pneumoniae, 8 patients with HAdV plus any other respiratory viral pathogen plus mycoplasma pneumoniae). Additionally, two patients with HAdV plus mycoplasma pneumoniae plus parainfluenza virus plus Streptococcus pneumoniae were excluded from statistical data of 174 cases as it is difficult to group.

Children with viruses-typical bacteria detected were younger than children with only HAdV detected; and children with viruses–atypical bacteria detected were older than other groups. Children with viruses-atypical bacterial pathogens detected were more likely to be girls than children with only HAdV or HAdV-viruses pathogens. The age distribution of children with HAdV-viruses co-infection most closely resembled those with HAdV infection alone. For 6–23 months age group, the number of children in viruses–typical bacteria group was significantly larger than that in single HAdV infection group. For 24–60 months period, the number of children in HAdV-virues and viruses–atypical bacteria group were more than that viruses–typical bacteria group (Table 2).

**Table 2:** Demographic and clinical characteristics of infected children

Characteristic	Single HAdV (*n* = 56)	HAdV- viruses (*n* = 79)	viruses-Typical Bacteria (*n* = 19)	viruses–Atypical Bacteria (*n* = 18)
Gender(male)	37(66.07)	56(70.90)	10(52.63)	7(38.90)^ce^
age (months) IQR	14(10–35.25)	12(8–25)	11(6–12)^b^	18.5(12–38.25)^ef^
Age group				
< 6	7(12.50)	10(12.66)	4(21.05)	0
6–23	25(44.64)	44(55.70)	14(73.68)^b^	10(55.56)
24–60	20(35.71)	21(26.58)	1(5.26)^bd^	5(27.78)^f^
> 60	4(7.14)	4(5.06)	0	3(16.67)^e^
Length of stay, days				
Median (IQR)	8.5(6–10)	8(6–10)	10(8–13)^bd^	7(6–8.25)^f^
Mean ± SD	8.52 ± 3.53	8.50 ± 4.53	10.68 ± 4.41	7.44 ± 2.41
Duration of fever (days)				
Median (IQR)	6(4–8)	5.5(3–7)	8(4.5–10.5)^d^	5(3–7.5)
Mean ± SD	6.94 ± 4.32	5.89 ± 4.77	8.00 ± 4.98	5.31 ± 2.96
Cough	56(100)	79(100)	19(100)	18(100)
Fever	49(87.50)	62(78.48)	14(73.68)	16(88.89)
Diarrhea	8(14.29)	15(18.99)	6(31.58)	2(11.11)
Wheezing	20(35.70)	37(46.84)	12(63.16)^b^	4(22.22)^f^
Vomiting	11(19.64)	15(18.99)	2(10.53)	2(11.11)
Outcome				
Disease severity of CPA				
Mild	25(44.64)	53(67.09)^a^	11(57.89)	17(94.44)^cef^
Severe	14(25.00)	14(17.72)	4(21.05)	1(5.56)
Very severe	17(30.36)	12(15.19)^a^	4(21.05)	0^c^
Admission to PICU	8(14.29)	19(24.05)	4(21.05)	0^e^
supplemental oxygen requirement	12(21.43)	18(22.78)	5(26.32)	0^f^
HAdV load from NPAs (log 10 copies/mL)	3.23 ± 1.87	3.25 ± 2.12	3.10 ± 2.16	2.60 ± 1.35

^a^*P* < 0.05, for comparison of single HAdV infections with combined virus-virus infections

^b^*P* < 0.05, for comparison of single HAdV infections with combined virus-typical bacterial infections

^c^*P* < 0.05, for comparison of single HAdV infections with combined virus-atypical bacterial infections

^d^*P* < 0.05, for comparison of virus-virus infections with virus-typical bacterial infections

^e^*P* < 0.05, for comparison of virus-virus infections with virus-atypical bacterial infections

^f^*P* < 0.05, for comparison of virus-typical bacteria with virus-atypical bacterial infections

For our cohort, clinical symptoms associated with CAP were generally similar among different infection groups. Besides, we also found that children with mix bacterial infections presented with a higher proportion of wheeze while compared with single HAdV infection or viruses-atypical bacteria infection. The duration of fever and length of stay in children with viruses-typical bacteria infections were significantly longer than those infected with HAdV-viruses infections (Table 2). Serious outcomes, including sever pneumonia, supplemental oxygen requirement, and admission to an intensive care unit were significantly less common in the viruses-atypical bacteria groups comparing with the other groups. Length of stay in the viruses-typical bacteria group was longer than all other groups (Table 2).

### Comparison of clinical manifestations between mild and severe HAdV pneumonia cases

In order to test the different between mild and severe HAdV pneumonia cases, comparison of clinical manifestations was undertaken. As shown in Table 3, successful HAdV typing was performed in 174 cases, the results showed no significant differences in clinical manifestations such as cough, wheezing, vomiting, and skin rashes between the mild pneumonia cases and severe pneumonia cases. However, in the severe pneumonia children, severe disease performances such as the shortness of breath, cyanosis, labored breathing, listless, antifeedant, and breathing machine support were noted. The incidence of diarrhea in children with severe pneumonia was higher than that in mild pneumonia children. Besides, compared with mild pneumonia children, children with severe pneumonia showed younger age, longer fever time, higher incidence of fever, and longer hospital stay. No significant difference was observed between the two groups in the percentage of white blood cells and neutrophils, erythrocyte sedimentation rate (ESR), and C-reactive protein (CRP).

**Table 3:** Comparison of clinical characteristics of HAdV infection with different severity of disease

	HAdV detection (*n* = 174)		×*2*/*t*/*Z*	*p*	Single HAdV detection (*n* = 70)		×*2*/*t*/*Z*	*p*
	mild(*n* = 108)	severe(*n* = 66)			mild (*n* = 38)	severe (*n* = 32)		
Gender (male)	63(58.33)	44(66.67)	1.201	0.273	20(52.63)	19(59.37)	0.302	0.572
Age(month)	16.00(11.0–33.75)	12.00(7.00–24.00)	−3.126	0.002	26.00(13.00–48.00)	12.00(8.50–15.00)	−3.817	0.000
Duration of Hospitalization (days)	7.00(6.00–9.00)	10.00(8.00–13.00)	−6.738	0.000	6.73(5.00–8.00)	10.00(9.00–12.00)	−5.004	0.000
Fever	81(75.00)	60(90.90)	6.746	0.009	30(78.94)	31(96.87)	3.512	0.061
Duration of fever (days)	3.00(0.75–6.00)	7.00(5.00–10.00)	−5.548	0.000	5.00(1.00–6.50)	8.00(6.00–10.00)	−4.081	0.000
WBC	8.26(6.62–11.78)	9.47(5.93–13.42)	−0.499	0.618	7.97(5.56–11.17)	8.34(5.53–10.30)	− 0.206	0.837
N%	43.29 ± 16.80	48.30 ± 17.48	−1.873	0.063	46.49 ± 15.53	52.23 ± 17.25	−1.466	0.147
Blood sinks	12.00(6.00–27.50)	13.00(5.00–24.00)	−0.009	0.992	12.00(8.50–30.00)	12.00(4.00–30.00)	−0.525	0.599
CRP	5.52(0.90–20.70)	5.79(1.70–19.40)	−0.900	0.368	7.62(1.14–18.69)	5.79(2.12–16.80)	−0.523	0.601
Mixed infection	70(64.81)	34(51.52)	3.013	0.083				
Wheezing	43(39.81)	32(48.48)	1.145	0.285	6(15.78)	18(56.25)	12.622	0.000
Vomiting	15(13.89)	15(22.72)	2.243	0.134	5(13.15)	6(18.75)	0.410	0.522
Diarrhea	13(12.03)	22(33.33)	11.56	0.001	4(10.52)	10(31.25)	4.663	0.031
Rash	8(7.40)	8(12.12)	0.726	0.394	3(7.89)	2(6.25)	0.000	1.000

As illustrated in Table 4, the clinical symptoms of mild pneumonia and severe pneumonia in 70 cases of single HAdV infection were found to be similar to the clinical symptoms of mild pneumonia and severe pneumonia in 174 cases of HAdV infection. That is to say, whether it is a single or mixed HAdV infection, severe adenovirus pneumonia is characterized by a small onset age, longer duration of fever, longer hospitalization, and prone to diarrhea. However, among HAdV single infection, children with severe pneumonia were more likely to have wheezing symptoms. According to adenovirus serotype, the 174 patients were divided into 5 groups. Among the children with severe pneumonia, HAdV-7 was more common, followed by HAdV-3. HAdV-1 and HAdV-2 were mostly found in children with light pneumonia. The average viral load of different serotypes were HAdV-1: 2.45 ± 1.89, HAdV-2: 2.66 ± 1.47, HAdV-3: 3.38 ± 2.17, HAdV-7: 3.78 ± 2.28, other types: 2.39 ± 1.55 (log10 copies/μL), and the difference was statistically significant (K-W test, *P* = 0.015). Pairwise comparison showed that the average viral load of HAdV-7 was higher than that of other types except the HAdV-3, and the difference was statistically significant. No significant difference was noted in other pairwise comparison.

**Table 4:** Comparison of mild and severe human adenovirus pneumonia

	HAdV detection (*n* = 174)		×*2*/*t*/*Z*	*p*	Single HAdV detection (*n* = 70)		×*2*/*t*/*Z*	*p*
	mild(*n* = 108)	severe(*n* = 66)			mild(*n* = 38)	severe(*n* = 32)		
HAdV-load	2.23(1.29–3.43)	4.05(2.58–6.35)	−6.226	0.000	2.24 ± 1.46	4.94 ± 2.57	−5.268	0.000
HAdV hexon sequence genotype			12.142	0.016			8.090	0.088
1	20(18.5)	6(9.1)			4(10.5)	1(3.1)		
2	21(19.4)	6(9.1)			7(18.4)	3(9.4)		
3	16(14.8)	10(15.2)			5(13.2)	2(6.3)		
7	37(34.3)	39(59.1)			16(42.1)	24(75.0)		
Other^*^	14(13.0)	5(7.6)			6(15.8)	2(6.3)		

^*^ HAdV-4, HAdV-5, HAdV-6, HAdV-14, HAdV-21, HAdV-57

### Analysis of HAdV load in the NPAs

To study the potential relationship between HAdV load and the severity of pneumonia, further analysis was performed. The mean time from disease onset to the beginning of PCR of respiratory tract samples was 6.5 days, and no significant difference was found in this mean time between severe pneumonia group (6.53 ± 1.80 days) and mild pneumonia group (6.44 ± 1.30 days) (*P* > 0.05). HAdV load of respiratory tract samples was measured and the results were represents as log10 DNA copies per μL samples. The highest and lowest HAdV load of the 174 pneumonia children were 10.88 (log10 copies/μL) and 0.02 (log10 copies/μL) respectively, and the average of HAdV load was 3.27 ± 2.15 (log10 copies/μL). The median viral load which was found in patients with HAdV infection alone was not higher than those who had co-infection with other respiratory viruses (*p* = 0.408).

Based on the time from clinical symptoms appearance of children to the collection of samples, we divided the children into two groups namely less than or equal to 10 days group and more than 10 days group. The mean HAdV load were 3.39 ± 2.20 and 2.25 ± 1.37 (log10 copies/μL) in less than or equal to 10 days group and more than 10 days group respectively. Significant difference in HAdV load between the two groups (*P* = 0.022) was revealed.

According to the severity of pneumonia, the 174 children were divided into mild pneumonia group (*n* = 108 cases) and severe pneumonia group (*n* = 66 cases). The HAdV load in the mild and severe pneumonia groups were 2.23 (1.29–3.43) and 4.05 (2.58–6.35) (log10 copies/μL) respectively, with significant difference in HAdV load between the two groups (*P* = 0.000). The 174 cases were further divided into mild (108 cases), severe (33 cases), and very severe (33 cases) subgroups, and the corresponding viral load were 2.44 ± 1.52, 5.07 ± 1.93, and 6.40 ± 2.29, respectively. For viral load comparison, the difference was statistically significant between each two groups (*P* = 0.000). Correlation analysis showed that HAdV virus load was positively correlated with the severity of pneumonia (*r* = 0.477, *P* = 0.000). Then, the 70 children with HAdV infection alone were also divided into mild pneumonia group (*n* = 38 cases) and severe pneumonia group (*n* = 32 cases) based on the severity of pneumonia. A high viral load was more prevalent among children diagnosed with severe diseases (*P* = 0.000). The result indicated that children with severe pneumonia had higher HAdV load.

### Correlation analysis of HAdV load and severity of pneumonia

In order to study the correlation of HAdV load and severity of pneumonia, further analysis was undertaken. For univariate analyses, multiple dichotomous and continuous variables were tested for their association with mild and severe pneumonia (Table 3). The severity of the disease was set as the dependent variable, while age, hospital stay, duration of fever, viral load and the types of HAdV were set as independent variables. Then severe adenovirus pneumonia was considered as an important dependent variable and its risk factors were analyzed by applying multivariate logistic. In the mild adenovirus pneumonia vs severe adenovirus pneumonia model, after adjusting for the effects of age and duration of fever, HAdV load significantly increased the risk of severe pneumonia (Tables 5 and 6). The types of HAdV and hospital stay were not significantly associated with disease severity.

**Table 5:** Analysis of influencing factors in children related to severity of HAdV infection

	OR*	95%CI		Wald	*p*
		Severity of pneumonia			
Age	0.972	0.949	0.995	5.481	0.019
HAdV load	1.933	1.480	2.524	23.367	0.000
HAdV hexon sequence genotype	1.106	0.928	1.318	1.267	0.260
Duration of fever	1.234	1.102	1.381	13.341	0.000
Diarrhea	1.847	0.640	5.336	1.287	0.257

^*^Adjusted gender

**Table 6:** Analysis of influencing factors in children with single HAdV infection

	OR^*^	95%CI		Wald	*p*
		Severity of pneumonia			
Age	0.961	0.927	0.997	0.945	0.033
HAdV load	2.221	1.312	3.758	8.839	0.003
Wheezing	4.615	0.702	30.337	2.533	0.111
Duration of fever	1.418	1.053	1.908	5.308	0.021
Diarrhea	4.570	0.541	38.573	1.949	0.163

^*^Adjusted gender

### Diagnostic efficiency of HAdV load to evaluate the severity of the disease

**Fig. 1:**
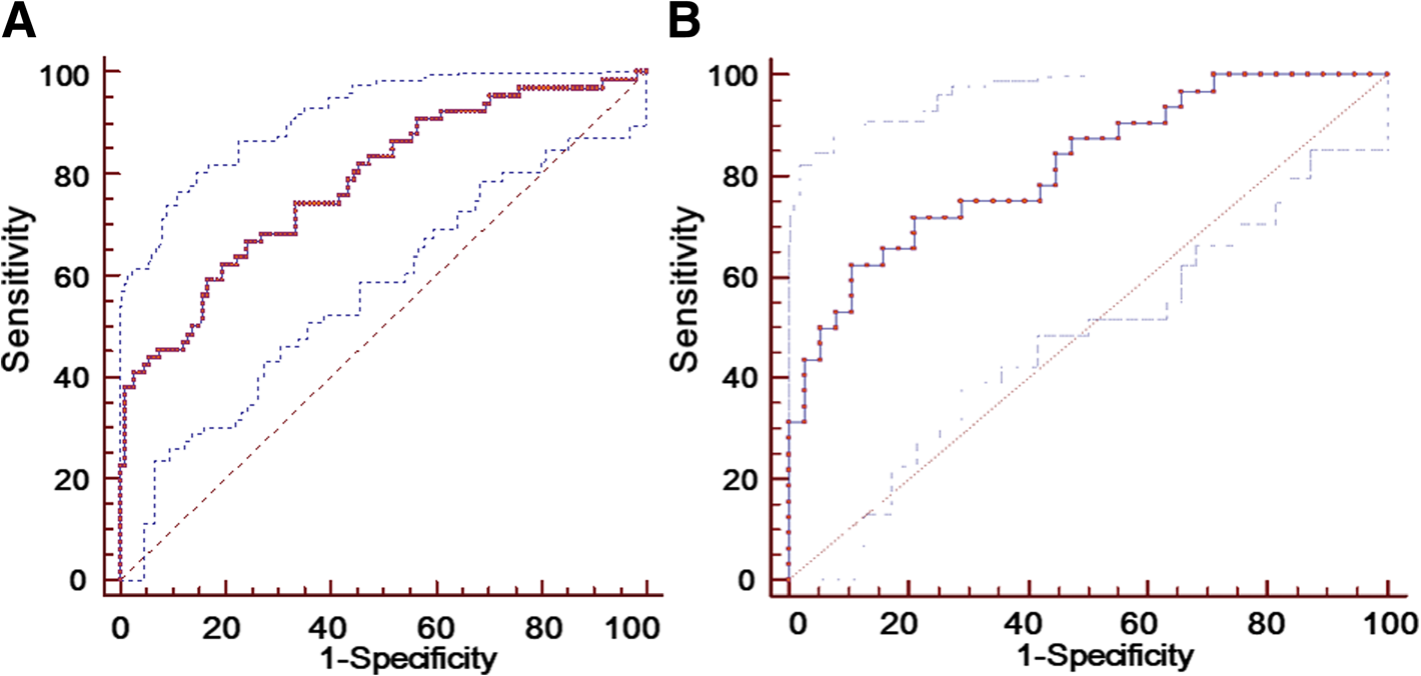
Receiver operating characteristic (ROC) curve of HAdV load to evaluate for the severity of HAdV infection (**a**) and single HAdV infection (**b**). The areas under the ROC curve (AUC) of HAdV load to predict severity of the disease were 0.782 (**b**) and 0.818 (**b**) respectively

To verify the diagnostic efficiency of HAdV load to evaluate the severity of the disease, receiver operating characteristic (ROC) curve analysis was used to analyze the accuracy of HAdV load as a biomarker of severity. The area under the ROC curve of 174 patients with HAdV infection and 70 patients with single HAdV infection were 0.782 (Fig. 1a) and 0.818 (Fig. 1b). These results suggested that HAdV load could be used as a predictor for the severity of childhood viral pneumonia.

## Discussion

In this study, we investigated the clinical manifestations of 174 pneumonia children infected by HAdV and the relationship between HAdV load and pneumonia severity. The results showed that pneumonia due to HAdV usually occurred in children with 6–23 months old and represented more prone to fever, longer duration of fever, longer disease course, and higher incidence of severe pneumonia compared with pneumonia children infected by non-adenovirus, which were consistent with previous studies [9, 18]. IgG antibodies to HAdV were demonstrated to be acquired transplacentally in over 90% infants at birth. These antibodies may protect the infants from the infection of HAdV when they are less than 6 months. Complement-fixing antibodies of HAdV types are only detected in 14% of infants at 6 months after birth. The phenomenon of the reduced of HAdV antibodies corresponds to the increased incidence of HAdV infection in children more than 6 months old [12]. In the current study, we found no significant differences in the clinical manifestations in cough, wheezing, vomiting, and diarrhea between pneumonia children infected by HAdV and the other viruses. Therefore, it is difficult to identify the infection of HAdV and other viruses in children at the early stages of pneumonia by clinical features. However, our study revealed significant differences between children with HAdV infection and those with other virus infection regarding the length of hospital stay, admission to the intensive care unit, oxygen requirements, and fatal outcome. If a child shows repeated high fever, long duration of fever, and the symptoms of respiratory infection, especially in children aged from 6 to 24 months old, pneumonia infected by HAdV should be taken into account. The limitation of this retrospective work was that for non-HAdV infection children, there was no clear distinction between other respiratory viruses infection. The interaction between specific viruses (e.g. adenovirus vs., rhinovirus and, adenovirus vs. respiratory syncytial virus) and identification of the independent effects of each virus increase understanding regarding the mechanisms of disease in viral coinfection. Previous studies reported that patients with HAdV infection commonly have a prolonged fever and a strong inflammatory response, resembling bacterial infections. Thus, on initial evaluation, it is difficult to distinguish adenoviral infections from severe bacterial infections [9]. This phenomenon may explain why so many antibiotics were prescribed. To date, the relationship between the clinical severity and infection status with single vs. multiple respiratory pathogens remains inconclusive. In our study, comparison of clinical characteristics of single HAdV and mixed bacterial infections revealed a longer duration of hospitalization in mixed bacterial infections, while oxygen therapy and the PICU admission rate did not differ between the two groups (Tables 1 and 2). This interesting phenomenon may illustrate the fact that multiple pathogen infection has a more effective mechanism for evading the innate immune response, resulting in slower pathogenic clearance. Further studies with larger populations comparing the severity and prognosis of CAP patients according to the type of virus as well as the different type of virus/bacteria combinations are needed.

Reports on the clinical impact of viral co-infection in patients with adenovirus infections are limited. Some investigators reported a lack of association between viral co-infections and clinical severity in children with HAdV infections [19]. In another retrospective study by Korea et al., co-infections with other respiratory viruses were frequent in children with HAdV infections less than 2 years of age [20]. We also found the clinical symptoms of both HAdV single infection and virus co-infection to be largely nonspecific. Similarly, a study from Peru and colleagues also found no higher prevalence of any clinical manifestations in co-infected patients than in those infected with HAdV alone [21]. Our results showed no significant differences in age, which was consistent with previous reports from Korea [20]. Coinfection with other respiratory viruses, on the other hand, did not lead to the severity of pneumonia, the duration of fever and hospitalization. In addition, admission to the intensive care unit, and oxygen requirements did not differ significantly between HAdV infection only and co-infection groups. These findings have the clinical implication that in patients with severe pneumonia, the contribution of HAdV should be considered with high priority, regardless of whether another respiratory pathogen has been detected.

Identifying HAdV serotypes is important in order to correlate the wide spectrum of clinical disease or symptoms with specific serotypes. The severity of pneumonia infected by HAdV is closely related to HAdV types. In China, the most common HAdV types were HAdV-3 and HAdV-7 [22]. HAdV-7 is more likely to cause severe pneumonia and represents highest lethality compared with other HAdV types in infants and children [18, 23]. In the present study, HAdV-3 and HAdV-7 were more frequently identified compared with other HAdV types, and HAdV-7 was the dominant serotype, accounting for 43.7% of children with HAdV infection. More than 50% of severe pneumonia children were infected by HAdV-7. Pneumonia caused by HAdV-7 was more severe compared with that caused by HAdV-1, HAdV-2, and HAdV-3, which was consistent with previous studies [18].

Currently, identification of adenovirus is usually performed for laboratory research and it is difficult to be implemented in clinic, therefore, it is difficult to judge the severity of pneumonia infected by HAdV based on type identification in clinic. RT-PCR can be used to perform qualitatively test for various viruses and quantify the viral load. This method will not increase additional economic costs and can contribute to clinical development.

In addition to HAdV types, the severity of the disease is also related to age and the immune status of the patients. The risk of severe pneumonia infected by HAdV is increased in lower than 7 years old patients, immunocompromised patients, or patients with chronic underlying disease or post-transplanting [24]. In a retrospective case-control study in Singapore, Rajkumar et al. assessed 85 hospitalized children diagnosed with HAdV infections in order to investigate the risk factors for severe infection [13]. They found that HAdV-7 was associated with more serious infections in children and young age (< 2 years) and significant comorbidities were associated with more severe HAdV infection [13]. However, there are no reports describing whether HAdV load is involved in severe respiratory disease in China. For diagnosing viral pneumonia, upper respiratory tract (URT) specimens have become the most common specimen type due to their logistical ease of collection. There are reports arguing that, for some viruses, a higher viral load in the URT is associated with worse outcomes [25–28]. A recently study found a high HAdV load in the respiratory tract on day 5–7 after disease onset and sustained viremia for 2 weeks or more, which may be associated with fatal clinical outcomes in adults [29]. Adenovirus viremia has been found in hematopoietic stem cell transplantation recipients and is associated with HAdV disease [30]. In this study, we carried out quantitative detection of HAdV, and then we combined the HAdV load with clinical symptoms to perform univariate analysis. Significant difference was noted in HAdV load between the patients with severe pneumonia and mild pneumonia. Further multivariate regression analysis of the severe and mild groups still showed that a high load of HAdV in the lower respiratory tract was associated with the development of severe disease. At the same time, age was an important factor that affected disease severity in the pneumonia group. Our study demonstrated that a high load of HAdV in the lower respiratory tract might be linked to the development of severe disease in children between 6 and 24 months. Additionally, it was illustrated that the types of HAdV were not statistically significant. The severe cases as well as the overall number of patients enrolled in this study might be relatively small, which, had a limiting effect on the statistical analyses. For example, it is possible that the comparison of pneumonia cases caused by HAdV-7 versus and other types would have statistical significance if the sample size was large enough.

Our study has two limitations. First, the number of analyzed patients was not large enough and more cases are needed to confirm our findings. Another limitation is that we did not conduct a study about viral load in whole blood, and the exact role of HAdV by themselves in pneumonia severity remains to be studied in the future. Third, our study did not monitor consecutive viral load in respiratory tract samples. Further studies are needed to verify the relationships between HAdV load and duration of the disease.

## Conclusion

HAdV is an important pathogen in Hunan, China. There was no specific feature for early HAdV pneumonia compared with other pneumonias. A high load of HAdV in the lower respiratory tract might be associated with pneumonia severity in children between 6 and 24 months. And HAdV load can help clinicians to identify severe cases.
